# Exploring the factors affecting the occurrence of postoperative MVI and the prognosis of hepatocellular carcinoma patients treated with hepatectomy: A multicenter retrospective study

**DOI:** 10.1002/cam4.6933

**Published:** 2024-01-29

**Authors:** Jilin Yang, Junlin Qian, Zhao Wu, Wenjian Zhang, Zexin Yin, Wei Shen, Kun He, Yongzhu He, Liping Liu

**Affiliations:** ^1^ The Second Clinical Medical College, Jinan University, Shenzhen Shenzhen China; ^2^ Department of Hepatobiliary Surgery Zhongshan People's Hospital (Zhongshan Hospital Affiliated to Sun Yat‐sen University) Zhongshan China; ^3^ Department of General Surgery The Second Clinical Medical College of Nanchang University, The Second Affiliated Hospital of Nanchang University Nanchang China; ^4^ Division of Hepatobiliary and Pancreas Surgery, Department of General Surgery The Second Clinical Medical College, The First Affiliated Hospital, Shenzhen People's Hospital, Jinan University, Southern University of Science and Technology Shenzhen China; ^5^ Division of Hepatobiliary and Pancreas Surgery, Department of General Surgery The First Clinical Medical College of Nanchang University, The First Affiliated Hospital of Nanchang University Nanchang China

**Keywords:** hepatectomy, hepatocellular carcinoma, microvascular invasion, prognostic factor

## Abstract

**Objective:**

To investigate the influencing factors affecting the occurrence of microvascular invasion (MVI) and the prognosis of hepatocellular carcinoma (HCC) patients treated with hepatectomy, and to explore how MVI affects prognosis in subgroups with different prognostic factors.

**Methods:**

Clinical data of a total of 1633 patients treated surgically for HCC in four treatment centers were included, including 754 patients with MVI. By using the Cox risk regression model and the Mann–Whitney *U*‐test, the common independent influences on prognosis and MVI were made clear. The incidence of MVI in various subgroups was then examined, as well as the relationship between MVI in various subgroups and prognosis.

**Results:**

The Cox risk regression model showed that MVI, Child–Pugh classification, alpha‐fetoprotein (AFP), hepatocirrhosis, tumor diameter, lymphocyte‐to‐monocyte ratio (LMR), and, Barcelona clinic liver cancer (BCLC) grade were independent determinants of overall survival (OS), and MVI, AFP, hepatocirrhosis, tumor diameter, and LMR were influencing determinants for disease‐free survival (DFS). The receiver operating characteristic (ROC) curve showed that MVI was most closely associated with patient prognosis compared to other prognostic factors. AFP, hepatocirrhosis, tumor diameter, and LMR were discovered to be common influences on the prognosis of patients with HCC and MVI when combined with the results of the intergroup comparison of MVI. After grouping, it was showed that patients with hepatocirrhosis, positive AFP (AFP ≥ 20 ng/mL), tumor diameter >50 mm, and LMR ≤3.4 had a significantly higher incidence of MVI than patients in other subgroups, and all four subgroups of MVI‐positive patients had higher rates of early recurrence and mortality (*p* < 0.05).

**Conclusions:**

MVI was found to be substantially linked with four subgroups of HCC patients with hepatocirrhosis, positive AFP, tumor diameter >50 mm, and LMR ≤3.4, and the prognosis of MVI‐positive patients in all four subgroups tended to be worse.

## Graphical abstract

This article explores the factors that influence the prognosis of patients with hepatocellular carcinoma and the occurrence of microvascular invasion (MVI), incorporates the common influences of both into the study, and combines previous studies to further elucidate how the included influences contribute to patient prognosis by affecting the occurrence of MVI.

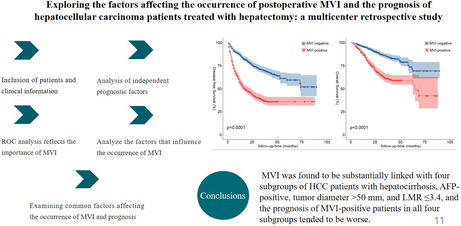

## 1 INTRODUCTION

Hepatocellular carcinoma (HCC) was one of the top three causes of cancer death in many countries.^1^ In China, liver cancer still has a high incidence and mortality rate, with new cases and fatalities making up more than half of all cases worldwide.^2^ Despite the fact that surgical treatment of HCC is now extensively developed and has become the most important therapy method for the radical treatment of HCC, patients still have a recurrence rate of more than 70% 5 years following surgery.^3^

One of the key elements affecting a patient's prognosis with HCC is microvascular invasion (MVI), and it is directly associated to recurrence and metastasis of HCC patients after surgery, according to study.^4^ MVI is the initial and crucial stage that leads to intrahepatic HCC spread and metastasis as well as the beginning of invasive HCC metastasis, all of which are detrimental to the prognosis of liver cancer.

After surgical resection of HCC, high recurrence rates and low long‐term survival remain, and MVI might be associated with the poor prognosis for HCC. However, there are not yet sufficient studies with large amounts of data to clarify which factors are more likely to lead to a higher incidence of MVI and a worse prognosis in patients with HCC. The purpose of the research was to use multicenter data to investigate the influencing factors affecting the occurrence of postoperative MVI and the prognosis of HCC patients treated with hepatectomy. To guide clinicians can screen for the high prevalence of MVI as early as possible and to give more personalized treatment plans in a timely manner, ultimately improving the prognosis of patients with HCC.

## 2 MATERIALS AND METHODS

### 2.1 Patients

Clinical data of patients with HCC from four medical centers between July 2015 and June 2022 were retrospectively analyzed. Inclusion criteria^1^: patients with HCC undergoing hepatectomy for the first time and not receiving other antitumor therapy prior to hepatectomy^2^; postoperative pathologic diagnosis of HCC with negative margins^3^; complete preoperative and follow‐up information. Exclusion criteria^1^: postoperative pathology suggestive of concomitant lymph node metastasis or extrahepatic metastasis^2^; severe organic pathology^3^; concurrent other malignant tumors. According to the aforementioned criteria, out of 1633 patients 305 cases from the Shenzhen People's Hospital, 591 cases from the Nanchang University's First Affiliated Hospital, 578 cases from the Nanchang University's Second Affiliated Hospital, and 159 cases from the Zhongshan People's Hospital were included in the study.

### 2.2 Hepatectomy resection and pathological diagnosis of MVI

Prior to hepatectomy, all patients have routine laboratory tests performed, including liver function tests, hepatitis virus infection testing, and tumor marker testing. Furthermore, imaging tests such as magnetic resonance imaging (MRI) and computed tomography (CT) are required to determine the preoperative tumor state.^5^ Under a microscope, MVI, a nested mass of cancer cells, can be observed in the artery lumen of endothelium‐covered arteries, and HCC is most commonly seen with portal vein branch invasion (including intraperitoneal vessels).^4^

### 2.3 Follow‐up

The disease‐free survival (DFS) rate was calculated from the date of hepatectomy through the date of tumor recurrence or the final date of follow‐up and the overall survival (OS) rate was calculated from the time when hepatectomy began to the date of death or the last follow‐up. New nodules in the liver, metastatic lesions inside or outside the liver, or the development of previously treated lesions are all considered postoperative recurrence. Early relapse was defined as tumor recurrence within 2 years after tumor resection, while late relapse was defined as tumor recurrence more than 2 years after initial surgery.^6^ After surgical resection, patients receive serum tumor markers as well as imaging exams every 2–3 months for the first 3 years and then every 6 months after 3 years. Patients who develop new liver lesions or locally recurrent lesions will again receive the appropriate antitumor therapy. The follow‐up period concluded on December 30, 2022.

### 2.4 Statistical analysis

The Shapiro‐Wilktest and Levene's test were used to identify all continuous variables. The Mann–Whitney *U*‐test, which is shown as the median (interquartile distance, IQR), was used to identify continuous data that had a non‐normal distribution; categorical data were expressed as number and percentages. For comparisons between these groups, the chi‐square test was used; univariate and multivariate Cox regression analyses were used to identify independent prognostic markers for DFS and OS, *p* < 0.05 indicated that the difference was statistically significant. The predictive value of each prognostic factor for patient prognosis was compared based on time‐dependent ROC curves. Kaplan–Meier (K–M) survival curves were used to analyze individual influencing factors and the relationship between MVI and prognosis. The above data were statistically analyzed by R software (Version 4.2.3).

## 3 RESULTS

### 3.1 Patient characteristics

**FIGURE 1:**
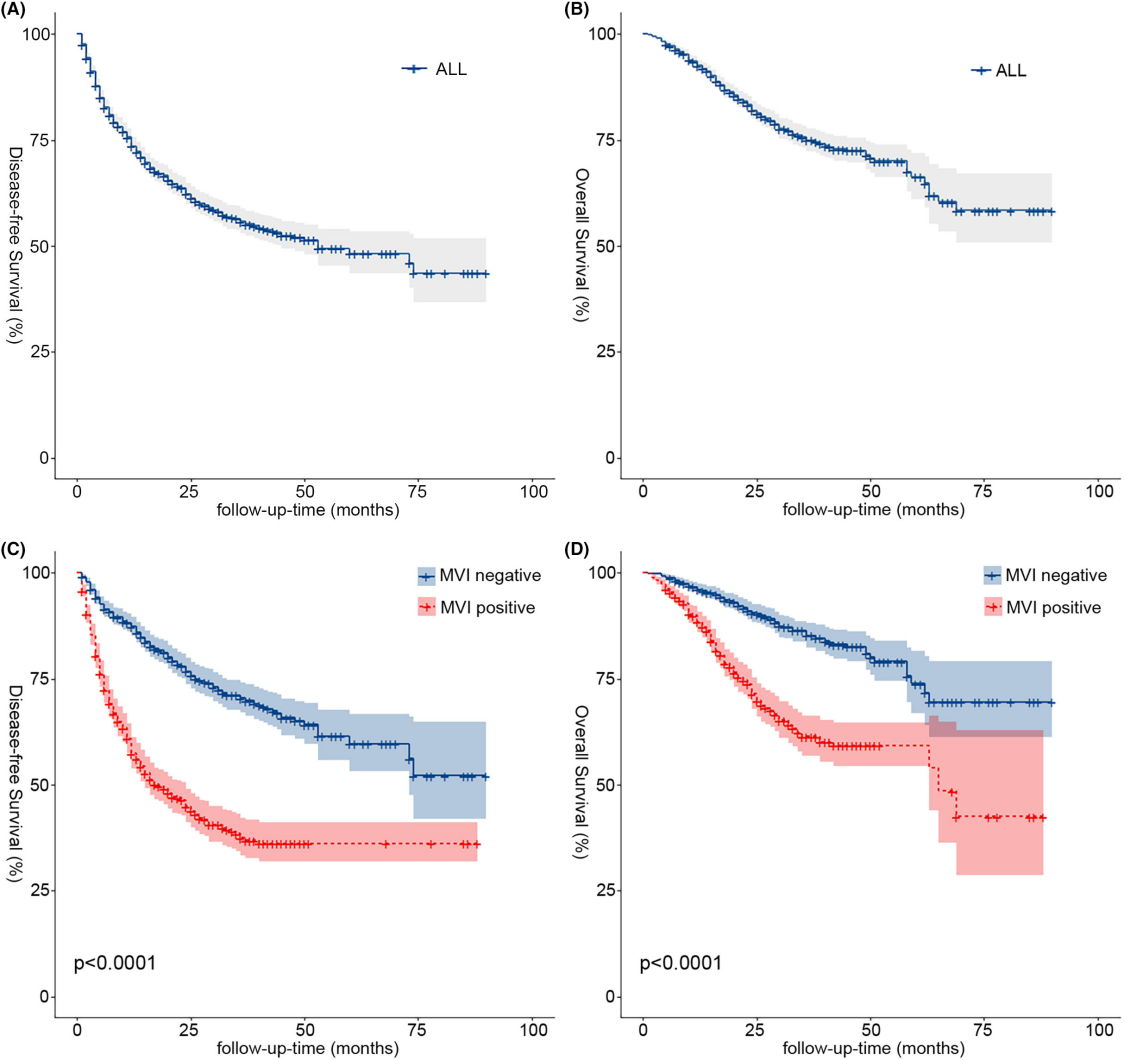
Kaplan–Meier analyses of DFS and OS for all patients (A, B) and MVI (C, D). The lightly stained areas on both sides of the curve are 95% CI ranges. DFS, disease‐free survival; MVI, microvascular invasion; OS, overall survival.

The study included 1633 patients, 1385 (84.8%) men and 248 (15.2%) women, with a median age as well as quartiles of 56 years (47–64 years), hepatocirrhosis in 1200 (73.5%) patients (Table 1), and a median follow‐up time of 23 months (1–90 months). By the time of follow‐up 621 patients had relapsed (550 early relapses) and 315 patients had died, with DFS at 1, 3, and 5 years of 73.6%, 55.8%, and 48.2%, respectively (Figure 1A); OS at 1, 3, and 5 years was 92.6%, 75.0%, and 66.3%, respectively (Figure 1B). MVI‐positive patients, of which there were 754 (46.2%), had lower 1‐, 3‐ and 5‐year DFS (57.4%, 37.4% and 36.2%) and OS (88.4%, 61.4% and 59.3%) than MVI‐negative 1‐, 3‐ and 5‐year DFS (87.3%, 70.5% and 59.6%) and OS (96.1%, 85.3% and 73.9%), with a statistically significant difference in prognosis (*p* < 0.05) (Figure 1C,D).

**TABLE 1:** Clinical characteristics of HCC patients between the MVI‐positive and MVI‐negative cohorts.

Variables		Total (*n* = 1633)	MVI		*p*‐value
			Positive (754, 46.2%)	Negative (879, 53.8%)	
Gender					
Male		1385 (84.8%)	664 (40.7%)	721 (44.1%)	0.0009
Female		248 (15.2%)	90 (5.5%)	158 (9.7%)	
Age (year)		56 (47–64)	55 (46–64)	57 (49–65)	0.0007
HBV					
Yes		1376 (84.3%)	641 (39.2%)	735 (46.1%)	0.4815
No		257 (15.7%)	113 (6.9%)	144 (8.8%)	
Cirrhosis					
Yes		1200 (73.5%)	574 (35.2%)	626 (38.3%)	0.0289
No		433 (26.5%)	180 (11.0%)	253 (15.5%)	
Child‐Pugh grade					
1		1563 (95.7%)	716 (43.8%)	847 (51.9%)	0.2044
2		70 (4.3%)	38 (2.3%)	32 (2.0%)	
AFP (ng/mL)					
Total		48.6 (6.0–718.0)	160.4 (11.9–1000.0)	20 (4.4–246.6)	<0.001
<20		664 (40.6%)	225 (13.8%)	439 (26.8%)	<0.001
≥20		969 (59.4%)	529 (32.5%)	440 (26.9%)	
ALT (U/L)		30.6 (22.0–45.0)	31.0 (23.0–48.0)	30.0 (21.0–44.0)	0.0082
AST (U/L)		34.7 (26.0–50.0)	39.0 (28.5–57.2)	32.0 (25.0–45.7)	<0.001
GGT (U/L)		52.1 (30.0–102.0)	65.2 (35.2–119.4)	43.0 (27.0–82.0)	<0.001
ALP (U/L)		95.0 (74.0–122.5)	100.1 (78.0–129.8)	91.0 (71.0–115.0)	<0.001
ALB (g/L)		41.1 (38.0–43.9)	40.8 (37.9–43.4)	41.4 (38.3–44.2)	0.0141
TBIL (μmol/L)		14.4 (10.7–19.7)	14.7 (10.9–19.3)	14.3 (10.6–19.9)	0.5845
PT (s)		11.9 (11.3–12.7)	12.0 (11.4–12.6)	11.9 (11.3–12.7)	0.2935
PLT (10^9^/L)		166 (121–215)	173 (127–227)	157 (114–208)	<0.001
WBC (10^9^/L)		5.34 (4.29–6.65)	5.44 (4.40–6.76)	5.27 (4.19–6.60)	0.0353
HGB (g/L)		141 (128–152)	143 (129–152)	140.0 (127–151)	0.0619
NLR		2.2 (1.6–3.2)	2.3 (1.7–3.3)	2.1 (1.5–3.0)	<0.001
LMR					
Total		3.4 (2.6–4.8)	3.3 (2.4–4.6)	3.6 (2.7–45.0)	0.0016
≤3.4		811 (49.7%)	402 (24.7%)	409 (25.0%)	0.0072
>3.4		822 (50.3%)	352 (21.5%)	470 (28.8%)	
PLR		109.2 (81.6–151.6)	116.7 (88.1–166.2)	102.6 (77.8–140.3)	<0.001
Tumor boundary					
Clarity		1151 (70.5%)	524 (32.1%)	627 (38.4%)	0.4496
No clarity		482 (29.5%)	230 (14.1%)	252 (15.4%)	
Tumor number (mm)					
Total		43 (27–70)	56 (35–85)	33 (22–55)	<0.001
≤20		227 (13.9%)	55 (3.4%)	172 (10.5%)	<0.001
20–50		735 (45.0%)	283 (17.3%)	452 (27.7%)	
>50		671 (41.1%)	416 (25.5%)	255 (15.6%)	
Tumor number					
1		1438 (88.1%)	636 (38.8%)	802 (49.1%)	<0.001
2		108 (6.6%)	55 (3.4%)	53 (3.2%)	
≥3		87 (5.3%)	63 (3.9%)	24 (1.4%)	
Relapse type					
No		1014 (62.1%)	359 (22.0%)	655 (40.1%)	<0.001
Late		69 (4.2%)	23 (1.4%)	46 (2.8%)	
Early		550 (33.7%)	372 (22.8%)	178 (10.9%)	

Abbreviations: AFP, alpha‐fetoprotein; ALB, albumin; ALP, alkaline phosphatase; ALT, alanine transaminase; AST, aspartate aminotransferase; GGT, gamma‐glutamyl transpeptidase; HBV, hepatitis B; HCC, hepatocellular carcinoma; HGB, hemoglobin; LMR, lymphocyte‐to‐monocyte ratio; MVI, microvascular invasion; NLR, neutrophil‐to‐lymphocyte ratio; PLR, platelet–lymphocyte ratio; PLT, platelets; PT, prothrombin time; TBIL, total bilirubin; WBC, white blood cells.

### 3.2 MVI influencing factors and Cox regression analyses

The 1633 HCC patients were classified into two groups: MVI positive and MVI negative. The comparative analysis revealed that the patients' gender, alanine aminotransferase (ALT), age, alkaline phosphatase (ALP), hepatocirrhosis, AFP level, aspartate aminotransferase (AST), gamma‐glutamyl transferase (GGT), albumin (ALB), platelets (PLT), white blood cell (WBC), lymphocyte‐to‐monocyte ratio (LMR), neutrophil‐to‐lymphocyte ratio (NLR), platelet‐to‐lymphocyte ratio (PLR), tumor diameter, and tumor number was statistically different between MVI subgroups (*p* < 0.05) (Table 1). The findings revealed that the proportion of hepatocirrhosis, AFP levels, and tumor diameter were higher in MVI‐positive patients than in MVI‐negative patients, although the LMR was lower in MVI‐positive patients than in MVI‐negative patients. Of the 550 HCC patients with early recurrence, 372 (67.6%) were MVI positive, while MVI‐positive patients accounted for 33.3% and 35.4% of patients with late recurrence and non‐recurrence, respectively, and the rate of early recurrence was much higher in the MVI‐positive group at 49.3% than in the MVI‐negative group at 20.1%, which was a statistically significant difference (*p* < 0.001) (Table 1).

We used both univariate and multivariate analyses to identify independent prognostic variables for DFS (Table 2). Univariate analysis showed that age, hepatocirrhosis, Child‐Pugh classification, AFP, ALT, AST, GGT, ALB, NLR, LMR, PLR, MVI, clarity of tumor border, tumor diameter, number of tumors, Barcelona clinic liver cancer (BCLC) stage, and MVI were associated with DFS (*p* < 0.05). Including these factors in the multifactorial analysis, the results showed that hepatocirrhosis (HR 1.25; 95% CI 1.03–1.52; *p* = 0.023), AFP (HR 1.00; 95% CI 1.00–1.00; *p* < 0.001), LMR (HR 1.01; 95% CI 1.00–1.02; *p* = 0.007), tumor diameter (HR 1.01; 95% CI 1.01–1.01; *p* < 0.001), and MVI (HR 2.02; 95% CI 1.69–2.42; *p* < 0.001) were independent prognostic variables for DFS.

**TABLE 2:** Univariate and multivariate survival analysis for disease‐free survival (DFS) in HCC patients.

Variables	Univariate analysis		Multivariate analysis	
	HR (95% CI)	*p*‐value	HR (95% CI)	*p*‐value
Gender	0.88 (0.70–1.10)	0.256		
Age (year)	0.99 (0.98–1.00)	0.005		
HBV (yes)	1.16 (0.92–1.45)	0.202		
Hepatocirrhosis (yes)	1.23 (1.02–1.47)	0.028	1.25 (1.03, 1.52)	0.023
Child‐Pugh grade (2)	1.44 (1.02–2.05)	0.041		
AFP (ng/mL)	1.00 (1.00–1.00)	0.000	1.00 (1.00, 1.00)	<0.001
ALT (U/L)	1.00 (1.00–1.00)	0.016		
AST (U/L)	1.00 (1.00–1.00)	0.000		
GGT (U/L)	1.00 (1.00–1.00)	0.000		
ALP (U/L)	1.00 (1.00–1.00)	0.120		
ALB (g/L)	0.97 (0.95–0.99)	0.000		
TBIL (μmol/L)	1.01 (1.00–1.01)	0.095		
PT (s)	1.04 (0.98–1.11)	0.170		
PLT (10^9^/L)	1.00 (1.00–1.00)	0.203		
WBC (10^9^/L)	1.00 (0.98–1.02)	0.776		
HGB (g/L)	1.00 (1.00–1.01)	0.119		
NLR	1.03 (1.00–1.05)	0.016		
LMR	1.01 (1.00–1.02)	0.037	1.01 (1.00, 1.02)	0.007
PLR	1.00 (1.00–1.00)	0.003		
MVI‐positive	2.99 (2.53–3.53)	0.000	2.02 (1.69, 2.42)	<0.001
Tumor boundary	0.81 (0.68–0.95)	0.012		
Tumor diameter (mm)	1.01 (1.01–1.01)	0.000	1.01 (1.01, 1.01)	<0.001
Tumor number				
1	Reference	–	–	–
2	1.51 (1.13–2.02)	0.005		
≥3	2.49 (1.87–3.31)	0.000		
BCLC grade				
0	Reference	–	–	–
1	2.23 (1.45–3.42)	0.000		
2	6.37 (4.07–9.96)	0.000		

Abbreviations: AFP, alpha‐fetoprotein; ALB, albumin; ALP, alkaline phosphatase; ALT, alanine transaminase; AST, aspartate aminotransferase; BCLC, Barcelona clinic liver cancer; GGT, gamma‐glutamyl transpeptidase; HBV, hepatitis B; HCC, hepatocellular carcinoma; HGB, hemoglobin; LMR, lymphocyte‐to‐monocyte ratio; MVI, microvascular invasion; NLR, neutrophil‐to‐lymphocyte ratio; PLR, platelet–lymphocyte ratio; PLT, platelets; PT, prothrombin time; TBIL, total bilirubin; WBC, white blood cells.

**FIGURE 2:**
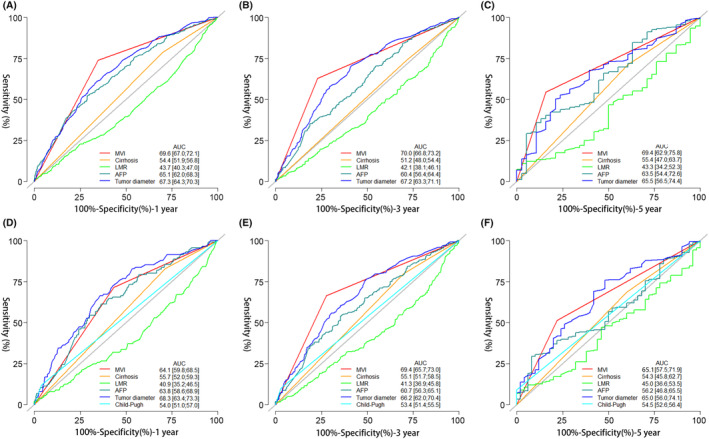
ROC analysis of DFS (A, B, C) and OS (D, E, F) at 1, 3, and 5 years for the Independent influences from multifactorial analysis of recurrence and death. AFP, alpha‐fetoprotein; AUC, area under the curve; BCLC: Barcelona clinic liver cancer; DFS, disease‐free survival; LMR, lymphocyte‐to‐monocyte ratio, OS, overall survival; ROC, receiver operating characteristic.

The independent prognostic variables for OS were also examined (Table 3). By univariate regression model analysis, we found that hepatocirrhosis, Child–Pugh classification, AFP, ALT, AST, GGT, ALP, ALB, TBIL, NLR, LMR, PLR, MVI, clarity of tumor border, tumor diameter, number of tumors, and BCLC stage were associated with OS (*p* < 0.05). Multifactorial analysis involving these variables showed that hepatocirrhosis (HR 1.61; 95% CI 1.20–2.16; *p* = 0.002), Child–Pugh classification (HR 2.12; 95% CI 1.32–3.38; *p* = 0.002), AFP (HR 1.00; 95% CI 1.00–1.00; *p* < 0.001), and LMR (HR 1.01; 95% CI 1.00–1.02; *p* = 0.010), tumor diameter (HR 1.01; 95% CI 1.01–1.01; *p* < 0.001), BCLC staging (HR 2.69; 95% CI 1.08–6.72; *p* = 0.034), and MVI (HR 2.37; 95% CI 1.83–3.06; *p* < 0.001) were independent prognostic factors for OS in patients with HCC. Using ROC curves, a comparison of independent prognostic determinants for DFS and OS found that MVI was most strongly linked with prognostic impact at 1, 3, and 5 years for DFS and at 3 and 5 years for OS, with the greatest area under the curve (AUC) (Figure 2).

**TABLE 3:** Univariate and multivariate survival analysis for overall survival (OS) in HCC patients.

Variables	Univariate analysis		Multivariate analysis	
	HR (95% CI)	*p*‐value	HR (95% CI)	*p*‐value
Gender	0.99 (0.73–1.35)	0.955		
Age (year)	1.00 (0.99–1.01)	0.728		
HBV	0.93 (0.69–1.25)	0.639		
Hepatocirrhosis (Yes)	1.62 (1.23–2.13)	0.001	1.61 (1.20–2.16)	0.002
Child‐Pugh grade (2)	2.79 (1.91–4.06)	0.000	2.12 (1.32–3.38)	0.002
AFP (ng/mL)	1.00 (1.00–1.00)	0.000	1.00 (1.00–1.00)	<0.001
ALT (U/L)	1.00 (1.00–1.00)	0.016		
AST (U/L)	1.00 (1.00–1.00)	0.000		
GGT (U/L)	1.00 (1.00–1.00)	0.000		
ALP (U/L)	1.00 (1.00–1.00)	0.000		
ALB (g/L)	0.95 (0.93–0.97)	0.000		
TBIL (μmol/L)	1.01 (1.00–1.01)	0.000		
PT (s)	1.06 (0.97–1.15)	0.194		
PLT (10^9^/L)	1.00 (1.00–1.00)	0.353		
WBC (10^9^/L)	1.00 (0.98–1.03)	0.740		
HGB (g/L)	1.00 (0.99–1.00)	0.585		
NLR	1.03 (1.00–1.06)	0.021		
LMR	1.01 (1.00–1.02)	0.015	1.01 (1.00–1.02)	0.010
PLR	1.00 (1.00–1.00)	0.002		
MVI‐positive	3.00 (2.37–3.80)	0.000	2.37 (1.83–3.06)	<0.001
Tumor boundary	0.73 (0.58–0.92)	0.008		
Tumor diameter (mm)	1.01 (1.01–1.02)	0.000	1.01 (1.01–1.01)	<0.001
Tumor number				
1	Reference	–	–	–
2	1.70 (1.15–2.51)	0.008		
≥3	2.45 (1.64–3.68)	0.000		
BCLC grade				
0	Reference	–	–	–
1	1.81 (1.01–3.23)	0.046		
2	5.85 (3.20–10.68)	0.000	2.69 (1.08–6.72)	0.034

Abbreviations: AFP, alpha‐fetoprotein; ALB, albumin; ALP, alkaline phosphatase; ALT, alanine transaminase; AST, aspartate aminotransferase; BCLC, Barcelona clinic liver cancer; GGT, gamma‐glutamyl transpeptidase; HBV, hepatitis B; HCC, hepatocellular carcinoma; HGB, hemoglobin; LMR, lymphocyte‐to‐monocyte ratio; MVI, microvascular invasion; NLR, neutrophil‐to‐lymphocyte ratio; PLR, platelet–lymphocyte ratio; PLT, platelets; PT, prothrombin time; TBIL, total bilirubin; WBC, white blood cells.

### 3.3 Joint analysis of prognostic influences and MVI

**FIGURE 3:**
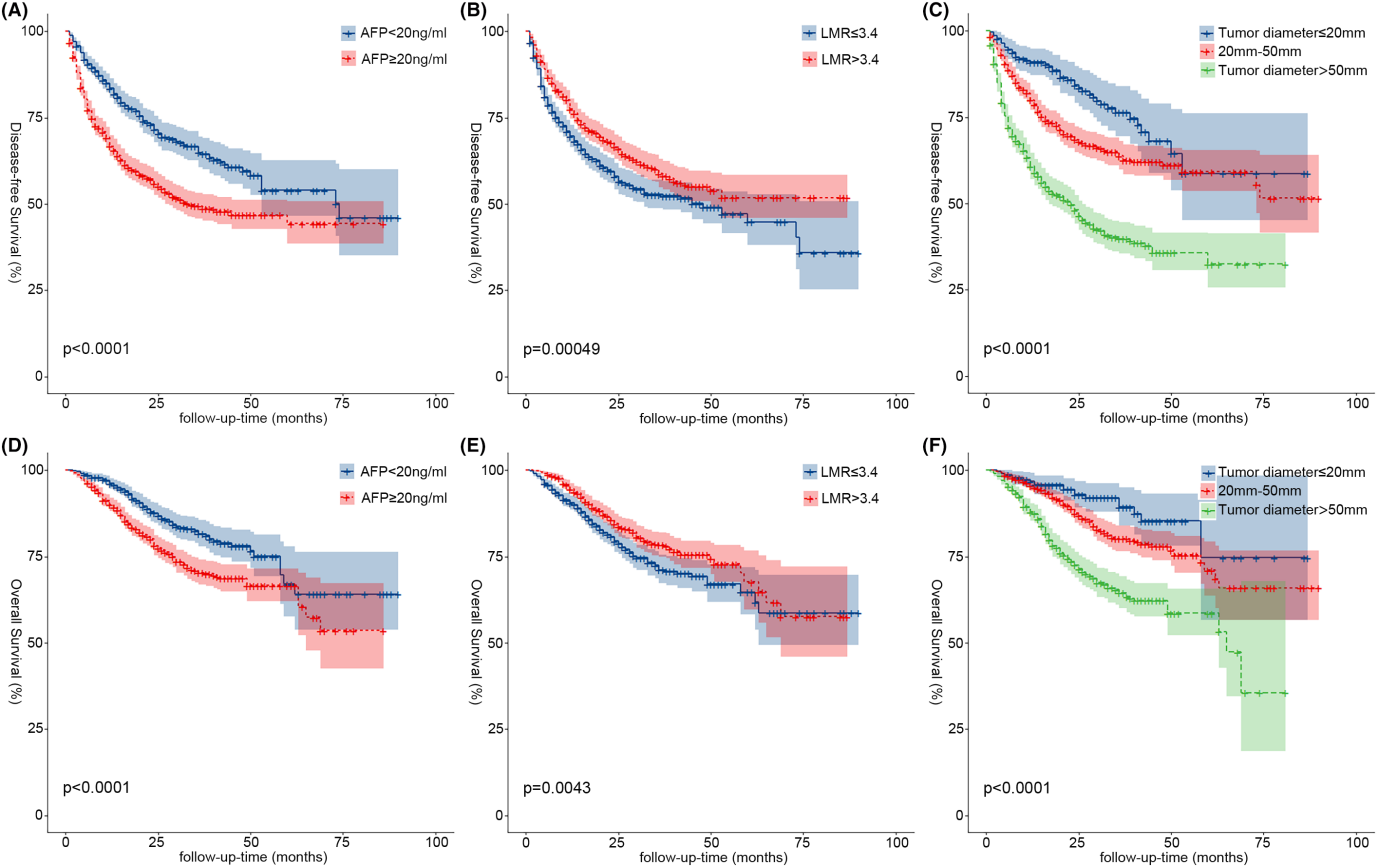
Kaplan–Meier analysis of DFS and OS for stratification of continuity risk factors (A, D, AFP, 20 ng/mL; B, E, LMR, 3.4; C, F, tumor diameter, 20 mm and 50 mm). AFP, alpha‐fetoprotein; DFS, disease‐free survival; LMR, lymphocyte‐to‐monocyte ratio; OS, overall survival.

To investigate the relationship between MVI and the prognosis of HCC, we included independent influences (AFP, hepatocirrhosis, tumor diameter, and LMR) common to the Cox regression models and stratified the continuous variables separately according to clinical significance: LMR was divided into two groups with median (LMR ≤3.4 and LMR >3.4); AFP with 20 ng/mL as the cutoff value was divided into the AFP‐positive group (AFP≥20 ng/mL) and AFP‐negative group (AFP < 20 ng/mL)^7^; tumor diameter was divided into three groups (tumor diameter ≤20 mm, tumor diameter between 20 and 50 mm, and tumor diameter >50 mm), and the K–M survival curves of each group were statistically significantly different (*p* < 0.001) (Figure 3). Using the Pearson chi‐square test, four independent prognostic factor subgroups were found to be statistically significantly different in the MVI subgroup (*p* < 0.001) (Table 1). The proportion of MVI‐positive patients with hepatocirrhosis, AFP‐positive, tumor diameter >50 mm, and LMR ≤3.4 was significantly higher than that of MVI‐negative patients. Additionally, we compared the association between MVI and prognosis in HCC patients within each influencing factor subgroup separately. We demonstrated a significant association between DFS and OS in HCC patients in all subgroups with the exception of those with tumor diameters ≤20 mm, where there was no significant association with OS in patients (*p* = 0.22) (Figure 6D).

## 4 DISCUSSION

HCC is a common and extremely malignant tumor with a high recurrence rate and a poor long‐term prognosis following hepatectomy.^8^ The study has shown that patients who have a positive pathological diagnosis of MVI after undergoing surgical resection of HCC are more likely to experience recurrence and have a lower long‐term survival rate.^9^ As a result, it is critical to improve patient prognosis by investigating the relationship between preoperative clinical data and MVI if it can diagnose MVI as early as possible before surgery and aid doctors in selecting the best treatment approach.

**FIGURE 4:**
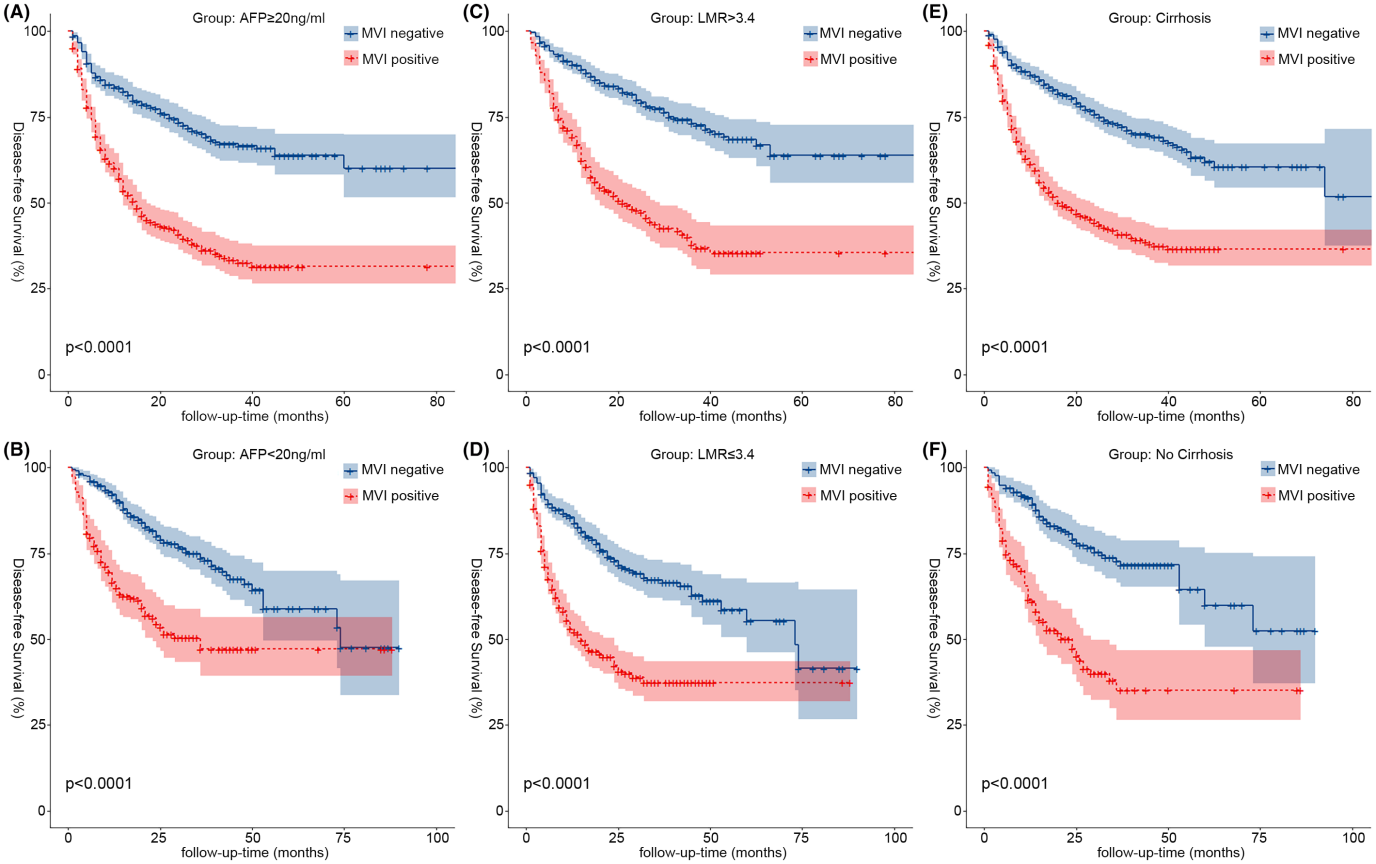
Kaplan–Meier analysis of DFS for MVI in different stratification of continuity risk factors (A, AFP‐positive (AFP ≥20 ng/mL); B, AFP‐negative (AFP <20 ng/mL); C, LMR >3.4; D, LMR ≤3.4; E, cirrhosis; F, No cirrhosis). AFP, alpha‐fetoprotein; DFS, disease‐free survival; LMR, lymphocyte‐to‐monocyte ratio; MVI, microvascular invasion.

**FIGURE 5:**
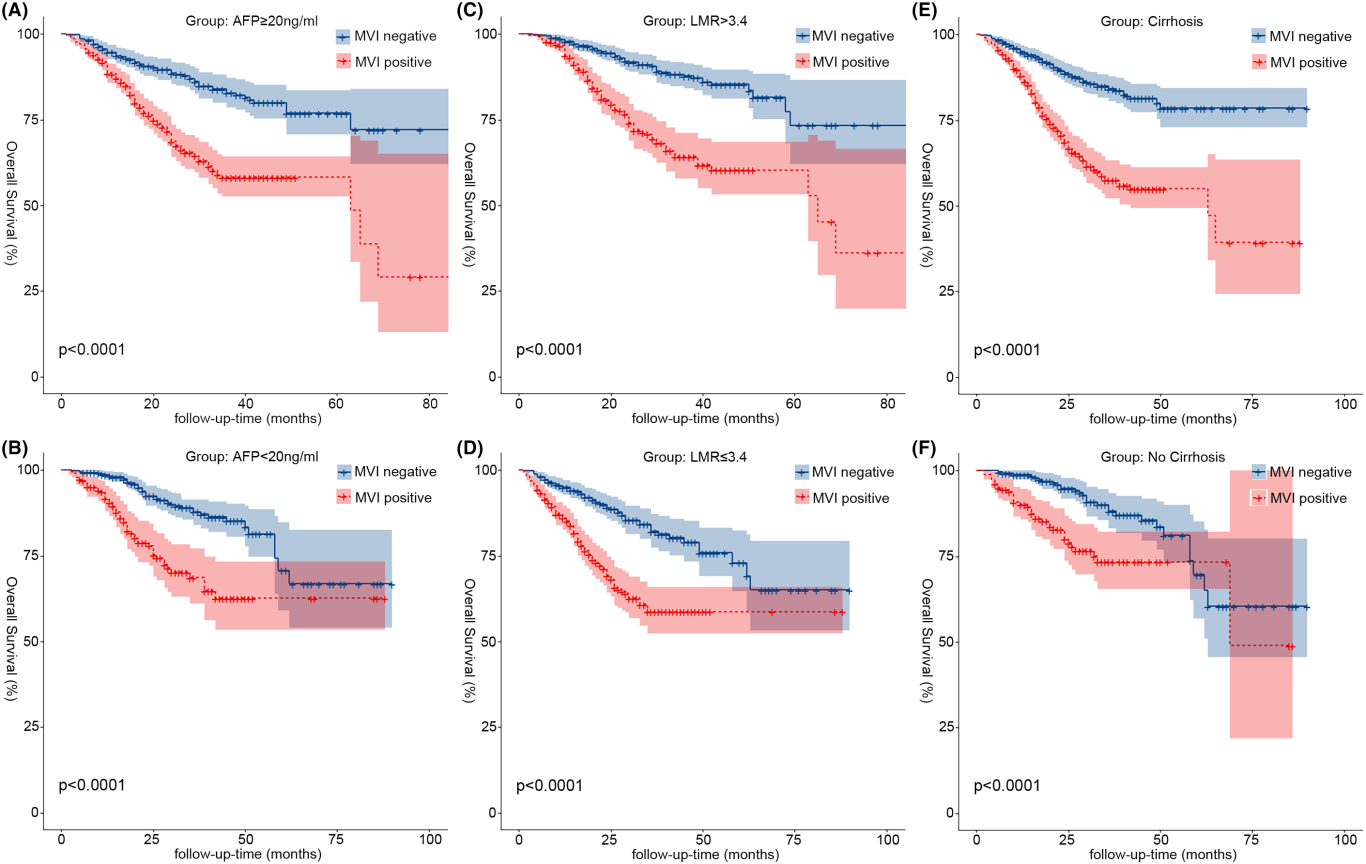
Kaplan–Meier analysis of OS for MVI in different stratification of continuity risk factors (A, AFP‐positive (AFP ≥20 ng/mL); B, AFP‐negative (AFP <20 ng/mL); C, LMR >3.4; D, LMR ≤3.4; E, cirrhosis; F, No cirrhosis). AFP, alpha‐fetoprotein; DFS, disease‐free survival; LMR, lymphocyte‐to‐monocyte ratio; MVI, microvascular invasion.

**FIGURE 6:**
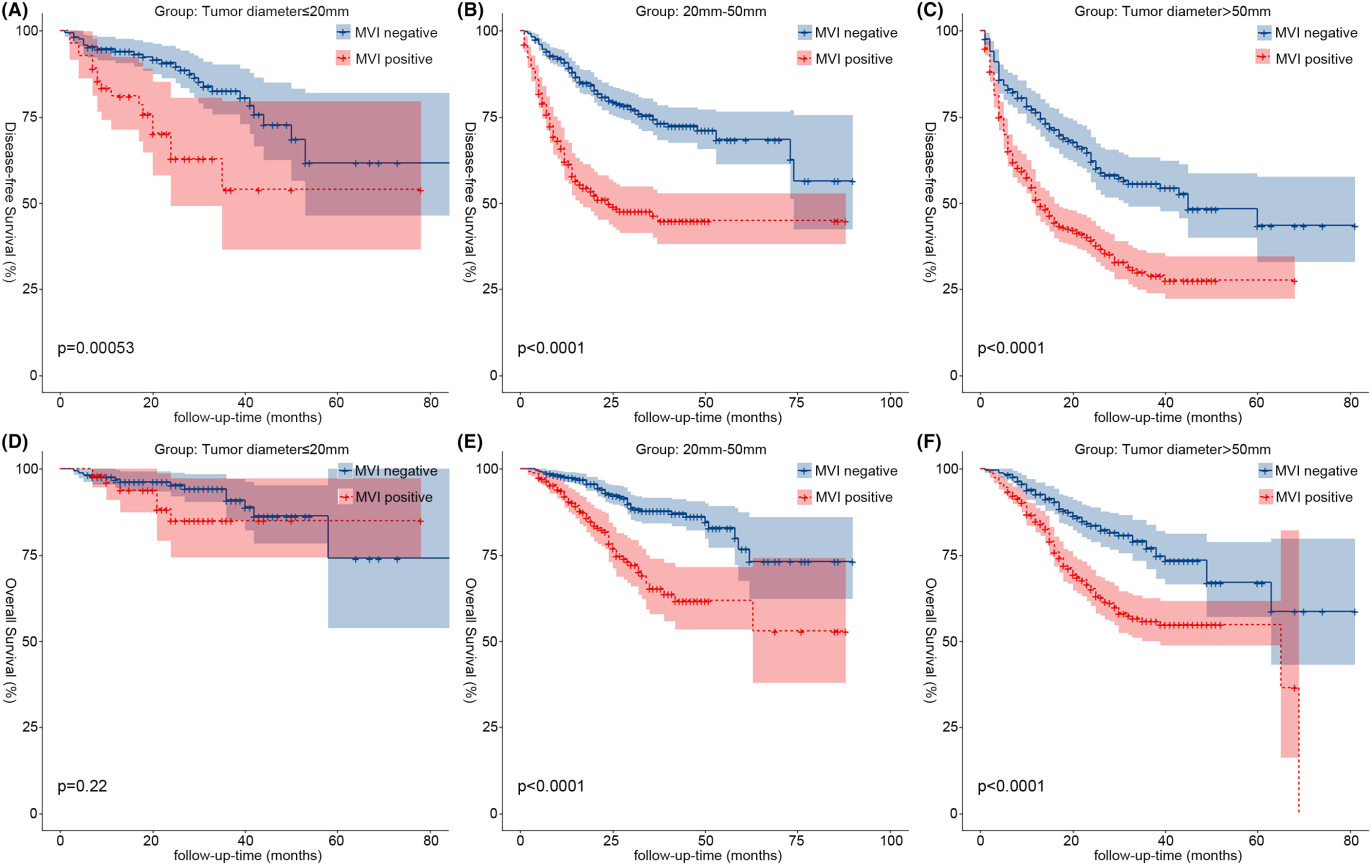
Kaplan–Meier analysis of DFS and OS for MVI in different stratification of tumor diameter (A,D, tumor diameter ≤ 20 mm; B,E, 20 –50 mm; C,F, tumor diameter > 50 mm). DFS, disease‐free survival; MVI, microvascular invasion; OS, overall survival.

We collected 1633 patients with HCC who had undergone hepatectomy from four treatment centers, of whom 754 were MVI positive, accounting for 46.2% of the total number of patients. We investigated not only the factors that influence MVI but also combined them with common independent risk factors for postoperative recurrence and death, and found that the proportion of MVI‐positive patients with hepatocirrhosis, positive AFP, tumor diameter >50 mm, and LMR ≤3.4 was higher than that of MVI‐negative patients. Furthermore, we observed that MVI was not significantly associated with OS in patients except in the tumor diameter ≤20 mm group (*p* = 0.22) (Figure 6D), and MVI was significantly associated with postoperative DFS (Figure 4) and OS (Figure 5) in all other subgroups (*p* < 0.001) especially in patients with early relapse and long‐term survival.

Hepatocirrhosis is caused by various mechanisms of liver injury that result in necrosis and fibrous formation; from a histological standpoint, it stands out for using nodal regeneration surrounded by dense fibrous septa, followed by parenchymal loss and collapse of the liver structure, all of which result in a considerable distortion of the hepatic vascular structure. This distortion will cause portal hypertension and hepatic synthetic dysfunction.^10^ The most significant risk factor for HCC, according to studies,^11^ is hepatocirrhosis of any cause, and in Western countries, more than 90% of HCC patients had hepatocirrhosis as an underlying condition. Savio G. Barreto et al. showed that hepatocirrhosis and MVI are important prognostic influences that jointly affect the prognosis of patients with HCC undergoing surgical resection, and that cirrhotic HCC patients also contribute to the formation of MVI due to concomitant repetitive inflammation and necrotic cell death, resulting in enhanced proliferation and the accelerated development of new HCC foci.^12^ In our investigation, hepatocirrhosis was not only an independent risk factor for postoperative death and recurrence in HCC patients, but it also had an impact on MVI. Of the 754 MVI‐positive patients in this study, 574 (76.1%) were cirrhotic, which was statistically higher than the 71.2% share of non‐cirrhotic patients. DFS at 1 year (56.1%) and OS at 1, 3, and 5 years (87.8%, 57.5%, and 55.0%) were significantly lower in the cirrhotic subgroup of MVI‐positive patients than in non‐cirrhotic patients DFS (61.6%) and OS (90.1%, 73.3%, and 73.3%), respectively, but the differences in DFS at 3 and 5 years were not significant, indicating that MVI positivity primarily affects early relapse and long‐term survival in cirrhotic HCC patients. After categorizing the type of recurrence, we found that 372 (67.6%) of the 550 HCC patients with early relapse were MVI positive, whereas 33.3% and 35.4% of the patients with late relapse and non‐relapse, respectively, were MVI positive, respectively, and it was further shown that the early relapse rate of 49.3% in the MVI‐positive patients was also much higher than that of 20.1% in the MVI‐negative group, which was statistically different (*p* < 0.001), further demonstrating that MVI is associated with early recurrence.^13^

In our investigation, LMR was included as a common influencing factor for prognosis and MVI in HCC patients. Numerous malignant disorders, including colon cancer, breast cancer, gastric cancer, and lung cancer, have been demonstrated to have poor prognoses when low LMR is present.^14^, ^15^, ^16^, ^17^ Decreased LMR is often caused by an increase in mononuclear macrophages, which have been shown to promote tumor progression in many malignant diseases, and their secretion of interleukin‐8 promotes tumor cell proliferation, invasive metastasis, and immune escape, thus increasing the likelihood of MVI, as well as being more likely to lead to early recurrence in patients after surgery.^18^, ^19^ There are no standardized criteria for the cutoff value of LMR, and in this study, we selected to stratify by the median LMR of 3.4 in this data to maximize generalizability and credibility as much as possible. The results of K–M survival curve analysis showed that patients in the LMR >3.4 group had better DFS (Figure 3B) and OS (Figure 3E) than those in the LMR ≤3.4 group (*p* < 0.05). LMR in MVI‐positive and MVI‐negative patients was statistically substantially different in both the ungrouped and the grouped conditions. MVI‐positive patients with LMR ≤3.4 (402, 53.3%) had a substantially greater proportion than MVI‐negative patients (409, 46.5%), and 49.6% of patients with LMR ≤3.4 were MVI‐positive, which was also higher than 42.8% of MVI‐positive patients with LMR >3.4. DFS (53.0%, 37.3%, and 37.3%) and OS (86.0%, 58.7%, and 58.7%) at 1, 3, and 5 years in MVI‐positive patients in the LMR ≤3.4 group were compared with those in MVI‐positive patients in the LMR >3.4 group (DFS: 62.3%, 37.9%, and 35.4%; OS: 91.1%, 64.2%, and 60.3%) were compared and the results were found to be similar to the analysis of the hepatocirrhosis subgroup, where the prognostic impact of low LMR levels on HCC patients was mainly seen in early relapse as well as in long‐term survival.

AFP is frequently reported to rise to very high levels with the development of HCC.^20^ However, studies have indicated that only 60%–70% of patients with HCC have increased AFP,^21^ and that hepatocirrhosis, hepatitis, and other malignancies impair the specificity of AFP.^22^ To separate the effect of MVI on different levels of AFP expression in our study, AFP was divided into the AFP‐negative (664, 40.6%) and AFP‐positive (696, 59.4%) groups at 20 ng/mL.^7^ We found that 70.2% of MVI‐positive patients were positive AFP, which was higher than the 50.0% of MVI‐negative patients, and that 54.6% of AFP‐positive patients were MVI positive, which was likewise much higher than the 33.9% of the AFP‐negative group. AFP reduced the activity of dendritic cells, natural killer cells, and T lymphocytes and enhanced the proliferation, cell motility, and invasiveness of HCC cell lines, thereby increasing the incidence of MVI.^23^ In our study, K–M survival analysis found a correlation between MVI and prognosis in AFP‐positive patients (*p* < 0.001). Tumor diameter was included in our study as a common influencing factor for survival prognosis and MVI. Studies^24^, ^25^ have shown that the size of the tumor is a significant risk factor for the emergence of MVI and postoperative recurrence of HCC. The incidence of MVI and postoperative recurrence in HCC patients increases progressively with increasing tumor diameter.^26^ Tumor size was separated into three groups in this study, with cutoff values of 20 and 50 mm. In terms of postoperative recurrence and mortality rates, as well as the percentage of MVI‐positive patients, patients with tumor sizes more than 50 mm exceeded the other two groups. Notably, our study indicated that patients with tumor diameters ≤20 mm had a much lower proportion of MVI‐positive patients than the other two groups, and the OS for the MVI subgroup showed no statistically significant difference (Figure 6D). It further suggests that the occurrence of MVI is highly associated to tumor size, with larger the diameter of the tumor, greater the risk of MVI, as well as higher the rate of postoperative recurrence and mortality.

## 5 CONCLUSION

In conclusion, by including and analyzing the association between prognostic‐related factors and MVI in a total of 1633 patients treated surgically for HCC in four treatment centers, our study found that hepatocirrhosis, AFP‐positive (AFP ≥20 ng/mL), tumor diameter >50 mm, and LMR ≤3.4 were strongly associated with the development of MVI, and also demonstrated that the long‐term prognosis and early relapse of HCC patients were both correlated with MVI. According to the above findings, in clinical work, for patients with preoperative LMR ≤3.4, cirrhosis, positive AFP, or preoperative imaging suggesting that the tumor diameter is greater than 50 mm, postoperative combination of further adjuvant therapeutic measures can be recommended to prevent the occurrence of MVI, and the frequency of follow‐up within 3 years after surgery should also be increased to detect early recurrence and give timely interventions to improve the prognosis of HCC patients.

## AUTHOR CONTRIBUTIONS

**Jilin Yang:** Conceptualization (equal); data curation (equal); formal analysis (equal); investigation (equal); methodology (equal); resources (equal); writing – original draft (equal). **Junlin Qian:** Conceptualization (equal); data curation (equal); investigation (equal). **Zhao Wu:** Data curation (equal); methodology (equal); resources (equal); software (equal). **WenJian Zhang:** Investigation (supporting); resources (supporting); software (supporting). **Zexin Yin:** Investigation (supporting); validation (supporting). **Wei Shen:** Conceptualization (equal); data curation (equal); methodology (equal); writing – review and editing (equal). **Kun He:** Conceptualization (equal); methodology (equal); supervision (equal); writing – review and editing (equal). **Yongzhu He:** Conceptualization (equal); data curation (equal); methodology (equal); resources (equal); supervision (equal); writing – review and editing (equal). **Liping Liu:** Conceptualization (equal); methodology (equal); supervision (equal); writing – review and editing (equal).

## FUNDING INFORMATION

This work was funded by Shenzhen Key Medical Discipline Construction Fund (Project Number: SZXK015), Guangdong Provincial and National Key Clinical Specialty Construction Project, and Basic and Applied Basic Research Foundation of Guangdong Province (Project Number: 2021A1515220059).

## CONFLICT OF INTEREST STATEMENT

All authors declare no conflict of interest.

## ETHICS STATEMENT

This retrospective study protocol was conducted in conformity with the Declaration of Helsinki (as revised in Edinburgh 2000) and was approved by the institutional ethics committee of the Shenzhen People's Hospital (Ethics number: LL‐KY‐2023064‐01), the First Affiliated Hospital of Nanchang University, Zhongshan People's Hospital, the Second Affiliated Hospital of Nanchang University. Due to the retrospective study design, individual consent for this retrospective analysis was waived.

## Data Availability

The datasets created and examined during the current work are not publically accessible due to privacy and ethical considerations, but they are available from the corresponding author upon justifiable request.
